# Relation of Social Participation Restrictions with Worsening Quality of Life in Japanese Patients with Behçet’s Disease: The 36-item Short Form Survey

**DOI:** 10.31662/jmaj.2024-0054

**Published:** 2024-11-18

**Authors:** Hideyo Tsutsui, Hiroaki Hoshino, Keiji Shiba, Taketoshi Fukasawa, Hirotoshi Kikuchi, Hiroko Oguchi, Takayoshi Ohkubo, Hajime Kono

**Affiliations:** 1 Faculty of Rehabilitation and Care, Seijoh University, Tokai, Japan; 2 Department of Hygiene and Public Health, Teikyo University School of Medicine, Tokyo, Japan; 3 Teikyo University Mizonokuchi Hospital, Kawasaki, Japan; 4 Teikyo University Hospital, Tokyo, Japan; 5 General Medical Education and Research Center, Teikyo University, Tokyo, Japan; 6 Division of Rheumatology and Allergy, Department of Internal Medicine, Teikyo University School of Medicine, Tokyo, Japan

**Keywords:** Behçet’s disease, Community life, Interpersonal interactions, Quality of life, 36-item Short Form Survey, Social participation

## Abstract

**Introduction:**

Patients with Behçet’s disease (BD) have a variety of symptoms, and the exacerbation of these symptoms affects their daily life and social participation and reduces their quality of life (QOL). This study aimed to clarify the relationship between social participation and QOL in BD patients.

**Methods:**

The BD-checklist 92 and 36-item Short Form Survey (SF-36) questionnaires were mailed to 10 affiliates. A total of 174 patients with BD completed the questionnaire. The patients were divided into two groups according to the presence or absence of problems in each “participation” category of the BD-checklist 92, and the SF-36 scores were compared. Subsequently, a correlational analysis was used to examine the relationship between the number of problem categories extracted from “participation” and scores on the eight subscales of the SF-36. Multiple regression analyses were performed to identify factors associated with SF-36 scores.

**Results:**

The SF-36 subscale scores were significantly lower in patients with problems in the participation category, particularly in those with difficulties in shopping, housework, relationships with friends and family, and community activities. A multiple regression analysis revealed that “basic interpersonal relationships” and “community life” were associated with the SF-36 subscales “role physical”, “social functioning”, “role emotional”, and “mental health”.

**Conclusions:**

This study showed that despite excluding the effects of BD-specific primary and secondary symptoms, problems with basic interpersonal relationships, such as those with friends and family, and restricted community activities were associated with reduced QOL.

## Introduction

The World Health Organization (WHO) defines quality of life (QOL) as “an individual’s perception of his or her position in life in the context of the culture and value systems in which he or she lives and in relation to his or her goals, expectations, standards, and concerns” ^(1)^. QOL is a broad concept influenced by an individual’s physical health, psychological state, independence, relationships within society, and relationships with salient environmental features ^(1)^. Social participation is a strong determinant of QOL ^(2)^, and environmental barriers are potential factors that affect health and QOL ^(3)^. Therefore, it is necessary to assess the QOL of patients by focusing not only on physical and psychological aspects but also on aspects such as social participation and the environment.

The three characteristics of Japanese patients with Behçet’s disease (BD) in recent years are as follows ^(4), (5), (6)^. First, the proportion of “complete” BD (patients with all four major symptoms of oral and genital ulcers, and eye and skin lesions) has decreased in recent years ^(4), (5)^. Secondly, the number of specific lesions is increasing ^(4), (5)^. Third, the proportion of older patients is increasing ^(6)^. Many patients can lead their daily lives without problems owing to the development of therapeutic drugs and advances in medication methods, dramatically improving treatment outcomes. However, the “Report on Public Health Administration and Services FY2022” ^(6)^ indicates that approximately 40% of Japanese patients with BD are older. BD is a chronic, relapsing disease of systemic inflammation that causes various symptoms in patients. In recent years, various age-related problems have arisen as patients age, in addition to the problems caused by the original symptoms of BD.

BD has various manifestations, including oral and genital ulcers, uveitis, erythema nodosum, and arthritis ^(7)^. Patients with BD experience numerous physical and psychosocial problems ^(8)^. Consequently, difficulties in daily living may restrict the social participation of patients with BD. However, to date, studies evaluating the QOL in patients with BD have only described the relationship between the main symptoms, BD-specific side symptoms, and QOL ^(9), (10), (11), (12)^. In a previous study, we reported that primary and secondary symptoms led to various limitations in daily life and social participation ^(8)^. However, we did not examine the relationship between social participation and QOL in patients with BD. A few other studies have examined this relationship ^(13)^. Based on the hypothesis that limited social participation decreases QOL in patients with BD, this study examined the relationship between QOL and social participation using the 36-item Short Form Survey (SF-36) ^(10)^ widely used to assess QOL. To assess “Social participation,” we used the BD-checklist 92 to identify physical and psychosocial problems associated with BD symptoms in Japanese patients with BD, based on the International Classification of Functioning, Disability and Health (ICF) ^(8)^. “Social participation” was classified into three categories. The first is participation in activities focusing on social connections, such as employment, schooling, community activities, hobbies such as sports, arts, and culture, and socioeconomic activities such as shopping. It includes shopping, school life, job, community life, hobbies, and leisure activities. The next is participation in personal interactions with family, neighbors, co-workers, and friends. It includes basic interpersonal, family, and married couple/lovers’ relationships. The last is participation in household activities such as meal preparation, cleaning, and laundry. It includes meal preparation and housework other than meal preparation.

The purpose of the present study was to determine the relationship between social participation and QOL in patients with BD using the results SF-36 and to identify the factors of social participation associated with QOL deterioration in patients with BD.

## Materials and Methods

### Data collection and procedures

A questionnaire using the Japanese SF-36 ^(15), (16)^ and BD-checklist 92 ^(8)^ was sent to 10 institutions that were members of the Behçet’s Disease Research Committee, Health, and Labor Sciences Research Grants (Research on Intractable Diseases) from the Ministry of Health, Labor, and Welfare of Japan. Supplementary Table 1 shows the names and contact details of the 10 collaborating institutions and the number of participants. The questionnaire was administered by primary care physicians (specialists who manage BD) to patients with BD who visited 10 medical institutions. The study was conducted between October 2019 and March 2020. Overall, 174 patients responded to the questionnaire. The exact response rate could not be determined because the number of patients for whom the questionnaires were distributed at each medical institution was unknown.

### Instruments

SF-36 ^(14)^ is a widely used instrument for assessing health-related QOL. The SF-36 measures eight subscales: 10 items for “Physical functioning (PF),” four items for “Role physical (RP),” two items for “Bodily pain (BP),” five items for “General health (GH),” four items for “Vitality (VT),” two items for “Social functioning (SF),” three items for “Role emotional (RE),” and five items for “Mental health (MH),” and two-component summary scores (physical component summary: PCS, mental component summary: MCS) that can be calculated from these eight subscales. The scores assigned to each category range from 0 to 100, with higher scores indicating better health and 0 and 100 representing the lowest and highest QOL, respectively. The scale was scored over the previous 4 weeks ^(14)^. Fukuhara et al. ^(15), (16)^ developed the Japanese version of this scale. Although the BD-QOL ^(17)^ is used to assess the QOL of patients with BD, a Japanese version of the BD-QOL has not yet been developed. Therefore, this study used the SF-36 ^(15)^, which is widely used to assess QOL and validated in Japanese version.

BD-checklist 92 was used in Japanese patients with to identify the physical and psychosocial problems associated with BD symptoms based on the ICF ^(8)^. This BD-checklist 92 has undergone validity and reliability assessments and demonstrated high validity and reliability ^(18)^. The BD-checklist 92 included the following categories from the ICF: 33 categories from the “Body functions” component, 8 categories from the “Body structures” component, 31 categories from the “Activities and participation” component, and 20 categories from the “Environmental factors” component ^(8)^. Of these, data from 10 categories corresponding to “Participation” were used for analysis (i.e., shopping; preparing meals; housework other than meal preparation; basic interpersonal relationships; family relationships; married couple/lovers’ relationship; school life; job; community life; and hobbies and leisure activities). The BD-checklist 92 checks whether a problem exists for each category. Therefore, the answer was either “yes or no.” By counting the number of categories in the BD-checklist 92 that were answered “problematic,” it is possible to assess which areas the patients with BD have many problems. The list of categories in the BD-checklist 92 is included in the Appendix (Supplementary Table 2).

### Statistical analysis

Of the 174 respondents, 23 had “suspected or possible BD” or were “unknown.” Therefore, these patients were excluded from the analysis. The 151 analyzed patients met the criteria for either complete or “complete BD” or “definitive BD,” which is the diagnostic standard of the Ministry of Health, Labor, and Welfare ^(19)^.

Patients with BD were divided into two groups according to whether they had problems in each “Participation” category of the BD-checklist 92. The 10 “Participation” categories used for the analysis were as follows: shopping, preparing meals, housework other than meal preparation, basic interpersonal relationships, family relationships, married couple/lovers’ relationships, school life, job, community life, hobbies, and leisure activities ^(8)^. The data included age, BD duration, number of problem categories in the “Participation” component, and scores on subscales of the SF-36 questionnaire, reported as mean ± standard deviation. Student’s t-tests were used to compare continuous variables. The chi-square test or Fisher’s exact test was used for categorical variables. Data included sex, BD type, specific lesions, and manifestations, reported as numbers and percentages. The chi-square test was used to compare the proportions. The Fisher’s exact test was used when the value of the expected variable was < 5. All symptoms included in the analysis were self-reported.

Additionally, to examine the relationship between BD-specific symptoms and participation categories, participants were divided into two groups according to the presence or absence of symptoms (oral and genital ulcers, eye and skin involvements, arthritis, depressive symptoms, and fatigue). Their scores were compared on the eight subscales of the SF-36 and two components of the scores. The analysis methods were similar to those used when the 10 participation categories were divided into two groups according to the presence or absence of problems.

Correlation analysis was subsequently used to examine the relationship between the number of problem categories extracted from the “Participation” component of the BD-checklist 92 and the scores on the eight subscales and two-component summaries of the SF-36. Spearman’s correlation coefficient was used to assess the correlations.

In addition, multiple linear regression analyses were performed to assess associations with SF-36 scores. First, we evaluated the relationship between the SF-36 scores and the number of problem categories extracted from each “Participation” category of the BD-checklist 92. The scores on the eight subscales (PF, RP, BP, GH, VT, SF, RE, and MH) and the scores on the two-component summaries (PCS and MCS) of the SF-36 were entered as independent variables multiple linear regression model, adjusted for age, sex, specific lesions (intestinal, neurological, and vascular), and manifestations (oral and genital ulcers, eye and skin involvement, arthritis, depressive symptoms, and fatigue), was constructed. Partial regression coefficients (B) were calculated to assess the association level and significance in the multiple regression analysis. *P*-values were presented without adjusting for multiple comparisons, in an exploratory manner. Associations with a *P*-value <0.05 were selected.

All statistical analyses were performed using the SPSS statistical software (SPSS Statistics Desktop for Microsoft Windows version 28.0; IBM, Armonk, NY, USA). Statistical significance was set at *P*-value < 0.05.

### Ethics

The Teikyo University Review Board approved this study (approval number:19-058). All patients consented to use the questionnaire for research purposes by checking the boxes in the questionnaire. This study was conducted under the tenets of the Declaration of Helsinki.

## Results

### Characteristics of the patients according to the presence/absence of problems in the “Participation” category

Table 1 shows the patient characteristics and SF-36 subscale and component summary scores according to the presence or absence of problems in the “Participation” category of the BD-checklist 92. In terms of symptoms, arthritis, depressive symptoms, and fatigue accounted for more than the group of patients who reported problems with almost all of the participation categories compared to the group of patients who reported no problems with those. In addition, patients with problems in “preparing meal,” “housework other than meal preparation,” “basic interpersonal relationships,” and “job” were more likely to have skin symptoms than those without these problems. Furthermore, patients with problems in “hobbies and leisure activities” such as sports, arts and culture, and travel were more likely to have eye involvement. Patients with problems in “basic interpersonal relationships” and “community life” had lower scores on all but BP than those without problems. Patients with problems in “married couple/lovers’ relationships” had lower scores on PF, SF, RE, and PCS than those without problems. Patients with “school life” problems had lower GH, VT, MH, and MCS scores than those without problems. Patients with “job” problems had lower RE, GH, SF, MH, and MCS scores than those without problems. Patients with problems with “hobbies and leisure activities” had lower scores on PF, RP, GH, SF, RE, MH, and MCS than those without problems.

**Table 1.** Characteristics of Patients by the Presence or Absence of Problems in Each “Participation” Category.

Characteristic	Shopping		Preparing meal		Housework other than meal preparation		Basic interpersonal relationships		Family relationships	
	Yes	No	Yes	No	Yes	No	Yes	No	Yes	No
	(n = 42)	(n = 109)	(n = 36)	(n = 115)	(n = 35)	n = 116)	(n = 43)	(n = 108)	(n = 30)	(n = 121)
Sex (male/female) [n]	13/29	58/51^*^	8/28	63/52^**^	7/28	64/52^**^	15/28	56/52	8/22	63/58^**^
Age (y)	53 ± 14	51 ± 14	53 ± 11	51 ± 14	55 ± 12	51 ± 14	49 ± 13	53 ± 14	50 ± 12	52 ± 14
Duration of BD (y)	14.7 ± 11.7	15.5 ± 11.8	14.7 ± 11.1	15.5 ± 12.0	15.9 ± 13.6	15.1 ± 11.2	15.0 ± 12.9	15.4 ± 11.4	13.7 ± 10.7	15.7 ± 12.0
Manifestation [n (%)]										
Oral ulcers	42 (100)	105 (96.3)	36 (100)	111 (96.5)	35 (100)	112 (96.6)	43 (100)	104 (96.3)	30 (100)	117 (96.7)
Genital ulcers	29 (69.0)	72 (66.1)	25 (69.4)	76 (66.1)	27 (77.1)	74 (63.8)	30 (69.8)	71 (65.7)	21 (70.0)	80 (66.1)
Eye involvement	31 (73.8)	78 (71.6)	26 (72.2)	83 (72.2)	24 (68.6)	85 (73.3)	31 (72.1)	78 (72.2)	21 (70.0)	88 (72.7)
Skin involvement	32 (76.2)	73 (67.0)	30 (83.3)	75 (65.2)^ *^	30 (85.7)	75 (64.7)^ *^	38 (88.4)	67 (62.0)^ **^	24 (80.0)	81 (66.9)
Arthritis	35 (83.3)	69 (63.3)^ *^	30 (83.3)	74 (64.3)^ *^	31 (88.6)	73 (62.9)^ **^	38 (88.4)	66 (61.1)^ **^	24 (80.0)	80 (66.1)
Depressive symptoms	33 (78.6)	32 (29.4)^ **^	26 (72.2)	39 (33.9)^ **^	25 (71.4)	40 (34.5)^ **^	33 (76.7)	32 (29.6)^ **^	21 (70.0)	44 (36.4)^ **^
Fatigue	35 (83.3)	60 (55.0)^ **^	30 (83.3)	65 (56.5)^ **^	31 (88.6)	64 (55.2)^ **^	36 (83.7)	59 (54.6)^ **^	26 (86.7)	69 (57.0)^ **^
Number of problem categories in ‘Participation’	6.1 ± 2.3	1.2 ± 1.3^**^	6.2 ± 2.3	1.5 ± 1.6^**^	6.3 ± 2.4	1.5 ± 1.6^**^	5.9 ± 2.5	1.3 ± 1.4^**^	6.1 ± 2.8	1.7 ± 1.9^**^
SF-36 score
Physical functioning	61.8 ± 26.9	84.9 ± 19.5^**^	62.9 ± 28.1	83.3 ± 20.5^**^	60.0 ± 28.0	84.0 ± 19.7^**^	64.8 ± 28.4	83.9 ± 19.8^**^	59.7 ± 27.4	83.1 ± 20.8^**^
Role physical	57.3 ± 30.1	75.9 ± 23.5^**^	56.9 ± 29.0	75.0 ± 24.6^**^	55.4 ± 31.1	75.3 ± 23.6^**^	54.5 ± 28.0	77.1 ± 23.4^**^	54.8 ± 29.8	74.6 ± 24.5^**^
Bodily pain	58.2 ± 24.2	65.9 ± 19.8^*^	62.1 ± 23.7	64.3 ± 20.6	57.7 ± 21.7	65.6 ± 21.0^*^	60.0 ± 20.6	65.3 ± 21.5	56.8 ± 23.4	65.5 ± 20.5^*^
General health	38.5 ± 19.7	48.5 ± 20.3^**^	39.0 ± 17.8	47.8 ± 21.0^*^	37.3 ± 19.7	48.2 ± 20.2^**^	36.1 ± 20.2	49.5 ± 19.6^**^	37.0 ± 18.3	47.8 ± 20.6^*^
Vitality	48.5 ± 13.9	52.7 ± 14.7	50.0 ± 12.8	52.0 ± 15.1	47.9 ± 12.8	52.6 ± 14.9^*^	46.1 ± 13.2	53.7 ± 14.5^**^	46.9 ± 14.8	52.7 ± 14.3^*^
Social functioning	61.0 ± 30.1	78.4 ± 24.0^**^	60.4 ± 28.9	77.7 ± 25.2^**^	60.0 ± 31.2	77.7 ± 24.2^**^	55.8 ± 27.7	80.7 ± 23.2^**^	59.6 ± 29.7	77.1 ± 25.2^**^
Role emotional	62.9 ± 30.5	76.9 ± 25.7^**^	65.1 ± 30.1	75.5 ± 26.6^*^	60.0 ± 30.0	76.9 ± 25.9^**^	57.0 ± 28.2	79.4 ± 24.9^**^	57.5 ± 32.3	76.9 ± 25.2^**^
Mental health	55.7 ± 21.3	64.5 ± 20.5^*^	59.0 ± 20.0	63.0 ± 21.4	53.9 ± 20.9	64.6 ± 20.5^**^	49.0 ± 20.2	67.3 ± 19.1^**^	52.5 ± 20.7	64.5 ± 20.5^*^
PCS	27.9 ± 24.9	45.8 ± 18.5^**^	27.4 ± 23.8	45.0 ± 19.6^**^	26.5 ± 24.7	45.1 ± 19.1^**^	27.4 ± 24.0	46.1 ± 18.6^**^	26.3 ± 23.8	44.4 ± 19.9^**^
MCS	45.1 ± 7.5	45.7 ± 8.8	46.3 ± 6.6	45.3 ± 8.9	44.8 ± 6.7	45.8 ± 8.9	42.4 ± 8.2	46.8 ± 8.2^**^	44.4 ± 7.0	45.9 ± 8.7
**Characteristic**	**Married couple/lovers’ relationships**		**School life**		**Job**		**Community life**		**Hobbies and leisure activities**	
	**Yes**	**No**	**Yes**	**No**	**Yes**	**No**	**Yes**	**No**	**Yes**	**No**
	**(n =24)**	**(n = 127)**	**(n = 3)**	**(n = 148)**	**(n = 90)**	**(n = 61)**	**(n = 33)**	**(n = 118)**	**(n =55)**	**(n = 96)**
Sex (male/female) [n]	8/16	63/64	2/1	69/79	37/53	34/27	10/23	61/57^*^	26/29	45/51
Age (y)	49 ± 13	52 ± 14	24 ± 5	52 ± 14^**^	49 ± 12	56 ± 14^**^	53 ± 14	51 ± 14	53 ± 14	51 ± 14
Duration of BD (y)	14.9 ± 12.8	15.4 ± 11.6	6.3 ± 3.1	15.2 ± 11.7	14.0 ± 11.0	17.2 ± 12.7	13.3 ± 10.9	15.9 ± 12.0	15.1 ± 12.2	15.4 ± 11.6
Manifestation [n (%)]										
Oral ulcers	24 (100)	123 (96.9)	3 (100)	144 (97.3)	89 (98.9)	58 (95.1)	33 (100)	114 (96.6)	54 (98.2)	93 (96.9)
Genital ulcers	17 (70.8)	84 (66.1)	1 (33.3)	100 (67.6)	63 (70.0)	38 (62.3)	19 (57.6)	82 (69.5)	34 (61.8)	67 (69.8)
Eye involvement	16 (66.7)	93 (73.2)	1 (33.3)	108 (73.0)	64 (71.1)	45 (73.8)	24 (72.7)	85 (72.0)	46 (83.6)	63 (65.6)^ *^
Skin involvement	18 (75.0)	87 (68.5)	3 (100)	102 (68.9)	72 (80.0)	33 (54.1)^ **^	24 (72.7)	81 (68.6)	38 (69.1)	67 (69.8)
Arthritis	20 (83.3)	84 (66.1)	3 (100)	101 (68.2)	71 (78.9)	33 (54.1)^ **^	27 (81.8)	77 (65.3)	43 (78.2)	61 (63.5)
Depressive symptoms	19 (79.2)	46 (36.2)^ **^	3 (100)	62 (41.9)	47 (52.2)	18 (29.5)^ **^	22 (66.7)	43 (36.4)^ **^	38 (69.1)	27 (28.1)^ **^
Fatigue	21 (87.5)	74 (58.3)^ **^	3 (100)	92 (62.2)	69 (76.7)	26 (42.6)^ **^	28 (84.8)	67 (56.8)^ **^	39 (70.9)	56 (58.3)
Number of problem categories in ‘Participation’	6.1 ± 2.7	1.9 ± 2.2^**^	6.3 ± 3.1	2.5 ± 2.7^*^	3.7 ± 2.7	1.0 ± 1.8^**^	6.3 ± 2.2	1.6 ± 1.8^**^	5.1 ± 2.7	1.2 ± 1.4^**^
SF-36 score										
Physical functioning	67.3 ± 26.0	80.6 ± 23.2^**^	65.0 ± 27.8	78.7 ± 24.0	76.0 ± 22.9	82.1 ± 25.4	59.1 ± 27.0	83.9 ± 20.2^**^	66.1 ± 26.6	85.5 ± 19.3^**^
Role physical	62.0 ± 27.0	72.3 ± 26.5	52.1 ± 26.0	71.1 ± 26.7	66.9 ± 24.9	76.2 ± 28.6^*^	50.2 ± 27.0	76.4 ± 23.8^**^	60.5 ± 28.2	76.6 ± 24.1^**^
Bodily pain	60.3 ± 21.2	64.4 ± 21.4	48.7 ± 20.8	64.1 ± 21.3	63.0 ± 22.1	64.9 ± 20.3	59.6 ± 24.1	64.9 ± 20.5	60.6 ± 24.0	65.6 ± 19.6
General health	41.8 ± 19.3	46.4 ± 20.8	43.0 ± 14.4	46.3 ± 20.2^**^	42.3 ± 20.1	50.6 ± 20.4^*^	33.8 ± 21.6	49.0 ± 19.1^**^	38.6 ± 20.3	49.7 ± 19.7^**^
Vitality	46.9 ± 15.7	52.4 ± 14.2	31.3 ± 6.3	51.9 ± 14.4^*^	49.7 ± 15.0	54.3 ± 13.4	44.7 ± 13.1	53.4 ± 14.4^**^	49.3 ± 15.3	52.8 ± 14.0
Social functioning	62.0 ± 27.7	75.8 ± 26.3^*^	54.2 ± 36.1	74.0 ± 26.7	69.3 ± 25.6	79.9 ± 27.8^*^	51.9 ± 26.4	79.7 ± 23.9^**^	61.6 ± 28.9	80.5 ± 23.3^**^
Role emotional	59.0 ± 28.6	75.7 ± 26.9^**^	50.0 ± 22.0	73.5 ± 27.7	71.0 ± 27.3	76.0 ± 28.3	55.1 ± 29.8	78.0 ± 25.0^**^	64.7 ± 29.5	77.8 ± 25.6^**^
Mental health	57.1 ± 20.0	63.0 ± 21.2	31.7 ± 7.6	62.7 ± 20.8^*^	58.3 ± 20.6	67.7 ± 20.7^**^	49.6 ± 17.2	65.6 ± 20.7^**^	57.6 ± 21.7	64.7 ± 20.3^*^
PCS	31.2 ± 23.0	42.6 ± 21.3^*^	33.7 ± 29.2	40.9 ± 21.8	38.5 ± 19.7	44.1 ± 24.6	23.3 ± 22.4	45.7 ± 19.2^**^	30.1 ± 22.3	46.9 ± 19.2^**^
MCS	44.6 ± 6.9	45.7 ± 8.7	31.8 ± 3.4	45.8 ± 8.3^**^	44.2 ± 8.2	47.6 ± 8.5^*^	42.9 ± 6.9	51.5 ± 13.6^*^	45.0 ± 8.0	45.9 ± 8.7

Differences in means and percentages in each variable between the groups that responded “problematic” and those that responded “not problematic” for each participating category on the “BD-checklist 92” were examined. Data on age, BD duration, number of problem categories in “participation”, and scores on dimensions of the SF-36 questionnaire are shown as mean ± standard deviation values. Student’s* t*‐tests were used to compare age, BD duration, number of problem categories in ‘Participation’, and scores on dimensions of the SF-36 questionnaire. Chi-squared tests were used to compare sex, BD type, specific lesions, and manifestations. SF-36, 36-item Short Form Survey; n, number; BD, Behçet’s disease; PCS, physical component summary; MCS, mental component summary **, *P* < 0.01; *, *P* < 0.05

### Characteristics of patients according to the presence of symptoms

Table 2 shows patient characteristics and SF-36 scores according to the presence or absence of 7 symptoms of BD. In the presence/absence of problems in the “Participation” category, patients with eye involvement were more likely to have problems with “hobbies and leisure activities” than those without eye involvement. Patients with skin involvement experienced more problems with “preparing meals” and “housework other than meal preparation,” “basic interpersonal relationships,” and “job” than those without. Patients with arthritis had more problems with “shopping,” “preparing meals,” “basic interpersonal relationships,” and “job” than those without arthritis. Patients with depressive symptoms were more likely than those without depressive symptoms to have problems in all areas except “school life.” Patients with fatigue were more likely than those without to have problems in all categories except “school life” and “hobbies and leisure activities.”

**Table 2.** Comparison of the Scores on Dimensions of the SF-36 Questionnaire between Patients with and without Each Symptom.

Characteristic	Oral ulcers		Genital ulcers		Eye involvement		Skin involvement		Arthritis	
	Yes	No	Yes	No	Yes	No	Yes	No	Yes	No
	(n = 147)	(n = 4)	(n = 101)	(n = 50)	(n = 109)	(n = 42)	(n = 105)	(n = 46)	(n = 104)	(n = 47)
Sex (male/female) [n]	68/79	3/1	38/63	33/17^**^	59/50	12/30^**^	41/64	30/16^**^	38/66	33/14^**^
Age (y)	53 ± 14	52 ± 14	56 ± 11	51 ± 14	52 ± 14	53 ± 14	48 ± 11	54 ± 15^*^	54 ± 13	51 ± 14
Duration of BD (y)	15.1 ± 11.7	17.4 ± 11.7	18.1 ± 10.7	15.3 ± 11.9	15.1 ± 11.0	16.3 ± 12.3	14.4 ± 10.7	16.1 ± 12.3	15.1 ± 11.3	16.3 ± 12.1
Manifestation [n (%)]										
Oral ulcers	147 (100)	0^**^	100 (99.0)	47 (94.0)	100 (99.0)	40 (95.2)	105 (100)	42 (91.3)^**^	103 (99.0)	44 (93.6)
Genital ulcers	100 (68.0)	1 (25.0)	101 (100)	0^**^	27 (77.1)	74 (63.8)	80 (76.2)	21 (45.7)^**^	79 (76.0)	22 (46.8)^**^
Eye involvement	105 (71.4)	4 (100)	66 (65.4)	43 (86.0)^**^	24 (68.6)	85 (73.3)	74 (70.5)	35 (76.1)	68 (65.4)	41 (87.2)^**^
Skin involvement	105 (71.4)	0^**^	80 (79.2)	25 (50.0)^**^	30 (85.7)	75 (64.7)^*^	105 (100)	0^**^	86 (82.7)	19 (40.4)^**^
Arthritis	103 (70.1)	1 (25.0)	79 (78.2)	25 (50.0)^**^	31 (88.6)	73 (62.9)^**^	86 (81.9)	18 (39.1)^**^	104 (100)	0^**^
Depressive symptoms	65 (44.2)	0	48 (47.5)	17 (34.0)	25 (71.4)	40 (34.5)^**^	53 (50.5)	12 (26.1)^**^	53 (51.0)	12 (25.5)^**^
Fatigue	95 (64.6)	0^*^	75 (74.3)	20 (40.0)^**^	31 (88.6)	64 (55.2)^**^	77 (73.3)	18 (39.1)^**^	80 (76.9)	15 (31.9)^**^
Number of problem categories in ‘Participation’	3.0 ± 2.9	1.9 ± 2.1*	3.6 ± 3.2	2.4 ± 2.6	2.7 ± 2.6	2.5 ± 2.8	3.3 ± 3.0	2.3 ± 2.6	3.6 ± 3.0	1.8 ± 2.2^**^
Participation categories
Shopping	42 (28.6)	0	29 (28.7)	13 (26.0)	31 (28.4)	11 (26.2)	32 (30.5)	10 (21.7)	35 (33.7)	7 (14.9)^*^
Preparing meals	36 (24.5)	0	25 (24.8)	11 (22.0)	26 (23.9)	10 (23.8)	30 (28.6)	6 (13.0)^ *^	30 (28.8)	6 (12.8)^ *^
Housework other than meal preparation	35 (23.8)	0	27 (26.7)	8 (16.0)	24 (22.0)	11 (26.2)	30 (28.6)	5 (10.9)^ *^	31 (29.8)	4 (8.5)
Basic interpersonal relationships	43 (29.3)	0	30 (29.7)	13 (26.0)	31 (28.4)	12 (28.6)	38 (36.2)	5 (10.9)^**^	38 (36.5)	5 (10.6)^**^
Family relationships	30 (20.4)	0	21 (20.8)	9 (18.0)	21 (19.3)	9 (21.4)	24 (22.9)	6 (13.0)	24 (23.1)	6 (12.8)
Married couple/lovers’ relationships	24 (16.3)	0	17 (16.8)	7 (14.0)	16 (14.7)	8 (19.0)	18 (17.1)	6 (13.0)	20 (19.2)	4 (8.5)
School life	3 (2.0)	0	1 (1.0)	2 (4.0)	1 (0.9)	2 (4.8)	3 (2.9)	0	3 (2.9)	0
Job	89 (60.5)	1 (25.0)	63 (62.4)	27 (54.0)	64 (58.7)	26 (61.9)	72 (68.6)	18 (39.1)^**^	71 (68.3)	19 (40.4)^**^
Community life	33 (22.4)	0	19 (18.8)	14 (28.0)	24 (22.0)	9 (21.4)	24 (22.9)	9 (19.6)	27 (26.0)	6 (12.8)
Hobbies and leisure activities	54 (36.7)	1 (25.0)	34 (33.7)	21 (42.0)	46 (42.2)	9 (21.4)^ *^	38 (36.2)	17 (37.0)	43 (41.3)	12 (25.5)
SF-36 score										
Physical functioning	75.7 ± 23.8	80.6 ± 24.5	71.7 ± 19.8	78.7 ± 24.7	77.5 ± 23.3	77.6 ± 24.7	77.4 ± 21.6	78.1 ± 25.3	66.2 ± 22.4	86.4 ± 21.6^**^
Role physical	64.7 ± 26.5	77.1 ± 23.8^*^	55.7 ± 23.7	71.9 ± 25.9^**^	64.7 ± 25.0	72.6 ± 26.7	64.5 ± 24.8	72.3 ± 26.7	56.7 ± 23.9	79.0 ± 23.7^**^
Bodily pain	58.6 ± 18.5	70.3 ± 21.9^**^	53.1 ± 16.2	64.7 ± 20.7^*^	62.3 ± 20.6	63.2 ± 20.4	58.2 ± 18.5	65.6 ± 21.1^*^	50.4 ± 14.9	72.5 ± 18.9^**^
General health	38.5 ± 19.7	51.0 ± 22.2^**^	38.1 ± 13.6	45.1 ± 21.6	37.9 ± 18.6	48.4 ± 21.0^**^	37.2 ± 17.1	47.9 ± 21.5^**^	35.9 ± 18.1	50.3 ± 20.4^**^
Vitality	49.6 ± 12.8	54.4 ± 15.7	46.4 ± 12.3	52.3 ± 14.2	52.3 ± 14.1	50.7 ± 14.0	47.4 ± 12.7	53.5 ± 14.1^*^	45.3 ± 12.3	56.1 ± 13.5^**^
Social functioning	69.4 ± 27.3	79.7 ± 24.0^*^	66.1 ± 30.4	74.5 ± 25.7	68.2 ± 27.9	76.8 ± 25.1	73.3 ± 25.1	73.7 ± 27.4	64.9 ± 25.6	79.6 ± 25.6^**^
Role emotional	67.3 ± 28.1	80.4 ± 25.6^*^	61.1 ± 25.5	74.2 ± 27.9^*^	69.4 ± 29.9	74.0 ± 26.3	68.8 ± 26.8	74.3 ± 28.3	61.3 ± 26.6	80.5 ± 26.0^**^
Mental health	58.0 ± 20.1	70.2 ± 21.8^**^	58.8 ± 16.0	62.9 ± 22.2	58.8 ± 20.5	64.8 ± 21.8	56.5 ± 20.4	65.3 ± 21.1^*^	56.5 ± 18.6	66.8 ± 22.4^**^
PCS	37.0 ± 22.5	44.5 ± 18.3	32.3 ± 23.0	41.3 ± 20.9	36.4 ± 20.5	42.3 ± 21.9	40.4 ± 20.8	40.1 ± 22.0	31.1 ± 21.1	46.6 ± 19.3^**^
MCS	44.1 ± 7.5	48.5 ± 9.5^**^	44.6 ± 7.3	45.8 ± 8.7	44.3 ± 8.4	46.5 ± 8.4	42.9 ± 7.9	47.0 ± 8.3^**^	43.8 ± 7.3	47.0 ± 9.1^*^
**Characteristic**	**Depressive symptoms**		**Fatigue**		
	**Yes**	**No**	**Yes**	**No**						
	**(n =65)**	**(n = 86)**	**(n = 95)**	**(n = 56)**						
Sex (male/female) [n]	24/41	47/39^*^	31/64	40/16^**^						
Age (y)	54 ± 12	51 ± 14	50 ± 13	54 ± 14						
Duration of BD (y)	17.3 ± 14.7	15.1 ± 10.8	15.4 ± 12.0	15.1 ± 11.4						
Manifestation [n (%)]										
Oral ulcers	65 (100)	82 (95.3)	95 (100)	52 (92.9)^*^						
Genital ulcers	48 (73.8)	53 (61.6)	75 (78.9)	26 (46.4)^**^						
Eye involvement	50 (76.9)	59 (68.6)	62 (65.3)	47 (83.9)^*^						
Skin involvement	53 (81.5)	52 (60.5)^**^	77 (81.1)	28 (50.0)^**^						
Arthritis	53 (81.5)	51 (59.3)^**^	80 (84.2)	24 (42.9)^**^						
Depressive symptoms	65 (100)	0^**^	54 (56.8)	11 (19.6)^**^						
Fatigue	54 (83.1)	41 (47.7)^**^	95 (100)	0^**^						
Number of problem categories in ‘Participation’	4.7 ± 3.1	2.0 ± 2.3^**^	3.4 ± 2.8	1.3 ± 2.1^**^						
Participation categories										
Shopping	33 (50.8)	9 (10.5)^**^	35 (36.8)	7 (12.5)^**^						
Preparing meals	26 (40.0)	10 (11.6)^**^	30 (31.6)	6 (10.7)^**^						
Housework other than meal preparation	25 (38.5)	10 (11.6)^**^	31 (32.6)	4 (7.1)^**^
Basic interpersonal relationships	33 (50.8)	9 (10.5)^**^	36 (37.9)	7 (12.5)^**^					
Family relationships	21 (32.3)	9 (10.5)^**^	26 (27.4)	4 (7.1)^**^						
Married couple/lovers’ relationships	19 (29.2)	5 (5.8)^**^	21 (22.1)	3 (5.4)^**^						
School life	3 (4.6)	0	3 (3.2)	0						
Job	47 (72.3)	43 (50.0)^**^	69 (72.6)	21 (37.5)^**^						
Community life	22 (33.8)	11 (12.8)^**^	28 (29.5)	5 (8.9)^**^						
Hobbies and leisure activities	38 (58.5)	17 (19.8)^**^	39 (41.1)	16 (28.6)						
SF-36 score										
Physical functioning	62.2 ± 27.2	82.5 ± 21.0^**^	73.2 ± 24.5	87.4 ± 20.7^**^						
Role physical	52.3 ± 23.2	74.8 ± 25.0^**^	61.1 ± 25.9	86.9 ± 19.4^**^						
Bodily pain	54.9 ± 22.3	65.6 ± 19.4^**^	57.0 ± 20.0	75.3 ± 18.6^**^						
General health	32.1 ± 18.0	47.9 ± 20.2^**^	39.7 ± 19.6	55.9 ± 18.2^**^						
Vitality	42.1 ± 11.9	54.2 ± 13.3^**^	46.5 ± 13.6	60.0 ± 12.0^**^						
Social functioning	52.9 ± 26.6	79.7 ± 23.4^**^	66.2 ± 27.4	86.2 ± 21.0^**^						
Role emotional	53.3 ± 23.2	78.1 ± 26.7^**^	65.9 ± 27.8	85.1 ± 23.3^**^						
Mental health	47.2 ± 20.2	66.9 ± 19.4^**^	56.9 ± 19.9	70.9 ± 20.1^**^						
PCS	26.2 ± 23.1	44.3 ± 19.3^**^	35.4 ± 23.0	49.9 ± 16.4^**^						
MCS	40.8 ± 8.3	47.1 ± 7.9^**^	43.5 ± 7.7	49.1 ± 8.5^**^						

Differences in means and percentages of each variable by the presence or absence of each BD-specific symptom were examined. Data on age, BD duration, number of problem categories in “participation”, and scores on dimensions of the SF-36 questionnaire are shown as mean ± standard deviation values. Student’s* t*‐tests were used to compare age, BD duration, number of problem categories in ‘Participation’, and scores on dimensions of the SF-36 questionnaire. Chi-squared tests were used to compare sex, BD type, specific lesions, and manifestations. SF-36, 36-item Short Form Survey; n, number; BD, Behçet’s disease; PCS, physical component summary; MCS, mental component summary **, *P* < 0.01; *, *P* < 0.05

The mean number of problem categories extracted from the “Participation” categories of the BD-checklist 92 was higher in patients with oral ulcers, arthritis, depressive symptoms, and fatigue (all *P* < 0.001).

Patients with oral ulcers had lower RP, BP, GH, SF, RE, MH, and MCS scores than those without. Patients with genital ulcers had lower RP, BP, and RE than those without ulcers. Patients with eye involvement had lower GH scores than those without. Patients with skin involvements had lower BP, GH, VT, MH, and MCS scores than those without skin involvements. Patients with arthritis, depressive symptoms, and fatigue had lower scores on all scales than those without arthritis or depressive symptoms (all *P* < 0.05).

### Spearman’s rank correlation coefficients

The relationship between the number of problem categories extracted in the “Participation” component of the BD-checklist 92 and the scores on the dimensions of the SF-36 questionnaire was analyzed (Table 3). The number of problem categories in the “Participation” component was significantly correlated with all dimensions of the SF-36 questionnaire (all *P* < 0.05). It was shown that the scores on each dimension of the SF-36 questionnaire decreased as the number of problem categories extracted in the “Participation” component increased.

**Table 3.** Spearman’s Rank Correlation Coefficients between the Number of Problem Categories in the “Participation” Component of the “BD-Checklist 92” and the Scores on the Dimensions of the SF-36 Questionnaire.

Dimension	Number of the problem category in the component of “Participation”	
	*r*	*P*
Physical functioning	−0.460	< 0.001
Role physical	−0.431	< 0.001
Bodily pain	−0.183	0.025
General health	−0.380	< 0.001
Vitality	−0.278	< 0.001
Social functioning	−0.453	< 0.001
Role emotional	−0.327	< 0.001
Mental health	−0.349	< 0.001
PCS	−0.457	< 0.001
MCS	−0.212	0.009

BD, Behçet’s disease; SF-36, 36-item Short-Form Survey;* r*, correlation coefficient; PCS, physical component summary; MCS, mental component summary

### Multiple regression analysis

Multiple regression analyses were performed to identify the factors associated with SF-36 subscale scores (Table 4-1 and 4-2). In a multiple regression analysis of the number of problem categories extracted from the “Participation” category of the BD-checklist 92 with SF-36 scores (Table 4-1, PF, RP, SF, and PCS (B = −3.606, −1.789, −2.571, and −2.597; all *P* < 0.05) were inversely correlated. In a multiple regression analysis of each participation category with SF-36 scores (Table 4-2), “shopping” was inversely correlated with BP (B = −2.622, *P* = 0.001). “Basic interpersonal relationships” were inversely correlated with RP, SF, RE, MH, and MCS (B = −10.953, −15.198, −14.634, −16.368, and −4.165, respectively; all* P* < .05). “Family relationships” were inversely correlated with PF (B = −13.634, *P* = 0.006). “Community life” was inversely correlated with RP, SF, RE, and MH (B = −12.359, −14.511, −13.995, −10.159; all *P* < 0.05).

**Table 4-1.** Multiple Regression Analysis for the SF-36 Scores and the Number of Problem Categories Extracted from Each “Participation” Category of the BD-Checklist 92.

Independent variable	Number of the problem category in the component of “Participation”		
	B	95% CI	*P*
Physical functioning	−3.606	−4.933 to −2.278	< 0.001
Role physical	−1.789	−3.323 to −0.255	0.023
Bodily pain	0.351	−1.065 to 1.766	0.625
General health	−1.133	−2.482 to 0.216	0.099
Vitality	0.034	−0.915 to 0.983	0.943
Social functioning	−2.571	−4.206 to −0.936	0.002
Role emotional	−1.368	−3.130 to 0.395	0.127
Mental health	−0.951	−2.354 to 0.452	0.182
PCS	−2.597	−3.838 to −1.356	< 0.001
MCS	0.148	−0.430 to 0.727	0.613

SF-36, 36-item Short Form Survey; BD, Behçet’s disease; PCS, physical component summary; MCS, mental component summary; B, partial regression coefficient; CI, confidence interval. Adjusting for age, sex, specific lesions (intestinal BD, neurological BD, and vascular BD), and manifestations (oral ulcers, genital ulcers, eye involvement, skin involvement, arthritis, depressive symptoms, and fatigue)

**Table 4-2.** Multiple Regression Analysis for Each “Participation” Category and the SF-36 Scores.

Independent variable	Shopping			Preparing meal			Housework other than meal preparation		
	B	95%CI	*P*	B	95%CI	*P*	B	95%CI	*P*
Physical functioning	−0.921	−12.931 to 11.088	0.880	3.529	−7.925 to 14.983	0.543	−5.267	−16.862 to 6.327	0.370
Role physical	7.323	−6.270 to 20.916	0.288	4.415	−8.549 to 17.380	0.502	−3.354	−16.477 to 9.769	0.614
Bodily pain	−2.622	−15.449 to 10.206	0.001	10.185	−2.049 to 22.420	0.102	−3.377	−15.762 to 9.007	0.590
General health	7.107	−4.962 to 19.176	0.246	2.992	−8.519 to 14.503	0.608	−4.423	−16.075 to 7.229	0.454
Vitality	2.926	−5.592 to 11.445	0.498	6.579	−1.546 to 14.704	0.112	−1.946	−10.170 to 6.278	0.640
Social functioning	8.163	−6.278 to 22.603	0.265	5.545	−8.228 to 19.317	0.364	−0.913	−14.855 to 13.028	0.897
Role emotional	10.590	−4.812 to 25.993	0.176	15.382	0.692 to 30.072	0.040	−9.910	−24.780 to 4.960	0.190
Mental health	5.193	−6.683 to 17.069	0.389	17.574	6.247 to 28.901	0.003	−9.513	−20.979 to 1.953	0.103
PCS	4.066	−7.098 to 15.230	0.472	1.981	−8.667 to 12.629	0.713	−2.490	−13.269 to 8.289	0.848
MCS	2.331	−2.731 to 7.394	0.364	5.599	0.771 to 10.428	0.023	−2.135	−7.022 to 2.753	0.389
**Independent variable**	**Basic interpersonal relationships**			**Family relationships**			**Married couple/lovers’ relationships**		
	**B**	**95%CI**	***P***	**B**	**95%CI**	***P***	**B**	**95%CI**	***P***
Physical functioning	−5.757	−15.303 to 3.789	0.235	−13.634	−23.312 to −3.956	0.006	6.225	−4.005 to 16.455	0.231
Role physical	−10.953	−21.758 to −0.149	0.047	−8.680	−19.634 to 2.274	0.119	12.190	0.611 to 23.769	0.039
Bodily pain	1.136	−9.060 to 11.332	0.826	−5.852	−16.189 to 4.486	0.265	6.051	−4.876 to 16.978	0.275
General health	−5.213	−14.806 to 4.380	0.284	−3.043	−12.769 to 6.683	0.537	8.267	−2.013 to 18.548	0.114
Vitality	−3.263	−10.034 to 3.508	0.342	1.005	−5.860 to 7.869	0.668	1.579	−5.677 to 8.835	0.668
Social functioning	−15.198	−26.676 to −3.720	0.010	−2.318	−13.956 to 9.319	0.694	5.040	−7.261 to 17.341	0.419
Role emotional	−14.634	−26.877 to −2.391	0.020	−6.341	−18.753 to 6.071	0.314	4.016	−9.104 to 17.136	0.546
Mental health	−16.368	−25.808 to −6.928	< 0.001	−2.585	−12.156 to 6.399	0.594	9.013	−1.104 to 19.130	0.080
PCS	−8.731	−17.606 to 0.143	0.054	−8.852	−17.849 to 0.145	0.054	5.996	−3.514 to 15.506	0.215
MCS	−4.165	−8.189 to −0.142	0.043	1.735	−2.345 to 5.814	0.402	1.985	−2.327 to 6.297	0.364
									
**Independent variable**	**School life**			**Job**			**Community life**		
	**B**	**95%CI**	***P***	**B**	**95%CI**	***P***	**B**	**95%CI**	***P***
Physical functioning	2.755	−21.067 to 26.578	0.819	0.731	−6.345 to 7.806	0.838	−5.727	−15.878 to 4.423	0.266
Role physical	8.524	−18.440 to 35.487	0.533	2.721	−5.287 to 10.729	0.503	−12.359	−23.848 to −0.870	0.035
Bodily pain	−3.864	−29.309 to 21.581	0.764	6.271	−1.286 to 13.829	0.103	1.745	−9.098 to 12.587	0.751
General health	−23.266	−47.206 to 0.674	0.057	−0.324	−7.435 to 6.786	0.928	−4.412	−14.613 to 5.789	0.394
Vitality	−9.624	−26.522 to 7.273	0.262	1.7830	−3.236 to 6.801	0.483	−6.900	−14.100 to 0.300	0.060
Social functioning	2.771	−25.873 to 31.416	0.849	−1.060	−9.568 to 7.447	0.806	−14.511	−26.716 to −2.306	0.020
Role emotional	6.065	−24.488 to 36.617	0.695	5.805	−3.269 to 14.879	0.208	−13.995	−27.013 to −0.976	0.035
Mental health	−1.547	−25.106 to 22.011	0.897	−1.325	−8.322 to 5.672	0.709	−10.159	−20.198 to −0.121	0.047
PCS	9.682	−12.464 to 31.828	0.389	1.260	−5.317 to 7.837	0.705	−7.835	−17.271 to 1.602	0.103
MCS	−6.940	−16.981 to 3.102	0.174	−0.398	−3.381 to 2.584	0.792	−3.105	−7.783 to 1.174	0.154
**Independent variable**	**Hobbies and leisure activities**								
	**B**	**95%CI**	***P***						
Physical functioning	−8.434	−17.383 to 0.515	0.065						
Role physical	−2.977	−13.106 to 7.152	0.562						
Bodily pain	−6.279	−15.828 to 3.280	0.196						
General health	−6.787	−15.780 to 2.206	0.138						
Vitality	0.063	−6.285 to 6.411	0.984						
Social functioning	−5.736	−16.496 to 5.025	0.294						
Role emotional	−1.193	−12.670 to 10.285	0.837						
Mental health	2.227	−6.623 to 11.077	0.619						
PCS	−6.334	−14.653 to 1.986	0.134						
MCS	−0.396	−3.376 to 4.169	0.836						

SF-36, 36-item Short Form Survey; BD, Behçet’s disease; PCS, physical component summary; MCS, mental component summary; B, partial regression coefficient; CI, confidence interval. Adjusting for age, sex, specific lesions (intestinal BD, neurological BD, and vascular BD), and manifestations (oral ulcers, genital ulcers, eye involvement, skin involvement, arthritis, depressive symptoms, and fatigue)

## Discussion

The present results showed that the QOL of Japanese patients with BD was significantly lower in the group with problems in the “Participation” category of the BD-checklist 92 we developed than those without problems. In particular, the QOL of patients with BD who had problems with relationships with friends, family, and community members and participation in community activities was significantly lower than that of patients without these problems. In addition, even after adjusting for the effects of BD-specific primary and secondary symptoms, problems with basic interpersonal relationships, such as friends and family, and restricted participation in community activities were associated with lower QOL.

In a study of adults over 50 years of age, Sieber et al. ^(20)^ showed that activities of daily living, instrumental activities of daily living (IADL), and depressive symptoms mediated the association between multimorbidity and QOL, with depressive symptoms being the most important (16.7 %). Furthermore, in a study of patients with spinocerebellar ataxia type 10, Santos et al. ^(21)^ reported that the worse the functional dependence on IADL, the worse the QOL in the PF domain. A similar trend was observed in the patients with BD who participated in the present study. In addition, patients with BD and symptoms of skin involvement, arthritis, depressive symptoms, and fatigue were more common in the group with these problems, which may be related to their decreased QOL. Arthritis in patients with BD causes pain and movement difficulties and affects social relationships; therefore, patients who have difficulty fulfilling their roles and responsibilities because of physical pain and movement difficulties have reduced QOL ^(22)^.

Similarly, patients with BD in the present study also experienced difficulties in shopping and housework due to functional impairment and movement disorders with arthritis, which made them feel burdened because they could not fulfill their roles in daily life, which may have led to a decrease in QOL. We also reported in this research when we developed the BD-checklist 92 that this fatigue can cause various restrictions in daily life, such as housework and/or daily activities ^(8)^. Therefore, patients with BD and these problems may have had significantly lower SF-36 scores than those without these problems. It has been previously reported that patients with BD and arthritis have lower SF-36 scores than patients with BD but without arthritis ^(10), (11)^.

Interpersonal and family problems increase psychological distress ^(23), (24)^. Maheri et al. ^(25)^ showed that interpersonal relationships were relatively strongly associated with QOL in a QOL study of patients with beta-thalassemia. In addition, Yohannes et al. ^(23)^ reported that anxiety was associated with difficulty in interpersonal relationships and chest symptom severity in a study of QOL in adult patients with cystic fibrosis (CF) and that one of the subdomains of CF-QOL, interpersonal relationships, was significantly correlated with anxiety and depression. The present study’s multiple regression analysis identified interpersonal relationships as factors associated with RP, SF, RE, MH, and MCS scores. A larger number of patients with BD and interpersonal and family relationship problems showed depressive symptoms than those without these problems. Therefore, depressive symptoms may accelerate a decline in QOL. Depressive symptoms would also affect social participation and QOL. In addition, the higher incidence of fatigue in patients with BD and problems in the category “basic interpersonal relationships” than those without problems may also be associated with a lower QOL. Fatigue was more common in patients with BD than in healthy controls ^(26), (27)^. A significant correlation between fatigue and poor QOL in patients with BD has been reported ^(28)^. Moses et al. ^(29)^ reported that patients with BD and arthritis had higher fatigue and functional impairment rates. In this study, patients with arthritis had a significantly higher rate of fatigue and significantly lower scores on all subscales and components of the SF-36 than those without arthritis. We also reported that the mental fatigue observed in patients might cause difficulties in communicating with their neighbors in a survey conducted during the development of the BD-checklist 92 ^(8)^. Arthritis can cause pain, swelling, and impaired movement. This renders them prone to fatigue while walking or otherwise moving about. This may have accelerated their depressive symptoms, leading to a decline in QOL.

If they cannot participate in varied community activities or their leisure activities, such as hobbies, are restricted, their QOL tends to decline ^(30), (31)^. For this study, “community life” is defined as participation in community-based activities such as those organized by neighborhood associations. Multiple regression analyses associated “Community life” with RP, SF, RE, and MH. Patients with rheumatoid arthritis with higher social participation restrictions experience more pain, fatigue, anxiety, and depression^(32)^. Similar results have been reported for patients with BD ^(22)^. Restrictions on participation in community events due to arthritis, depressive symptoms, and fatigue as well as restrictions on hobbies and leisure activities, may have contributed to the deterioration of QOL in the present study. Patients with depressive symptoms ^(33), (34)^ and fatigue ^(35), (36)^ are commonly diagnosed with BD. Depressive symptoms and fatigue are important factors affecting QOL. Uguz et al. ^(35)^ reported that major depression negatively impacted the QOL in patients with BD and that QOL negatively correlated with the severity of depressive symptoms in a study from Turkey. Canpolat et al. ^(22)^ showed that patients with BD who reported that fatigue affected their daily lives had lower mean scores for PF, RP, RE, VT, BP, and GH than those who did not report fatigue. In the present study, patients with depressive symptoms and fatigue had significantly lower scores on all subscales and components of the SF-36 than those without these symptoms. Additionally, patients with BD with depressive symptoms and fatigue were significantly more likely to have problems with “community life” than those without these symptoms. Patients with depressive symptoms were also significantly more likely than those without depressive symptoms to have problems with “hobbies and leisure activities.” Based on the results of the aforementioned previous studies and the results obtained in this study, the group of patients with BD who had problems in the “community life” and “hobbies and leisure activities” categories in the present study had more depressive symptoms and fatigue than the group of patients who did not have these problems, which may contribute to the decline in the QOL.

In addition, the present study revealed that the lower the restrictions on social participation, the lower the QOL. Patients with BD and various manifestations of BD showed lower SF-36 scores, suggesting that the manifestations of these various BD symptoms may have led to restrictions in social participation. Restrictions in social participation due to the manifestations of various symptoms of BD may have led to a lower QOL. Patients with BD have been reported to have lower QOL because of disease activity ^(37)^. However, in previous studies, reports on various symptom manifestations leading to reduced QOL did not mention restrictions on social participation. The results obtained in this study indicate that the greater the restrictions on social participation, the lower the QOL. This is not a relationship in which the main symptoms of BD directly reduce the QOL of patients, as has been said in the relationship between the main symptoms of BD and QOL, but a relationship in which the main symptoms of BD prevent social participation in varied activities such as hobby activities and interpersonal relationships, suggesting that the relationship is indirect, in that the main symptoms of BD interfere with varied activities such as hobbies and interpersonal relationships, and the restrictions of social participation decrease QOL.

According to the ICF, social participation may contribute to a sense of QOL ^(38)^. In addition, participation in social activities has been reported to have a significant positive association with QOL ^(39)^. Participating in social activities gives meaning to life ^(40)^. This indicates that social participation may improve QOL. To this end, it is important for health professionals, such as physicians, nurses, and therapists, not only to treat the typical symptoms of BD but also to consider whether patients with BD are forced to give up social participation, including hobbies and interpersonal relationships, due to the various symptoms of BD. In addition, from the perspective of patient care, medical social workers would also need to participate in team medicine to determine whether patients with BD are forced to give up social participation such as hobbies, leisure activities, and interpersonal relationships, and intervene to solve the problem.

### Limitations

The present study had several limitations. First, BD disease activity was not assessed using a questionnaire. Therefore, as BD disease activity was not investigated in the questionnaire, the effect of BD disease activity on QOL was not considered. BD severity may be a confounding or mediating variable. In addition, as the symptoms were self-reported, the possibility that depressive symptoms and fatigue were subjective could not be ruled out.

Second, considering the possibility that the comorbidity of various BD-specific symptoms may be confounding and mediating variables, multiple regression analysis was performed in the present study using BD-specific primary and secondary symptoms as adjustment variables, as well as age and sex. However, regarding the raw score of the SF-36, it cannot be denied that the comorbidity of BD-specific primary and secondary symptoms (especially depressive symptoms) is a confounding and mediating variable.

Third, no specific family relationships were indicated in response to the “family relationships” question. Therefore, it is unclear with which family members the “family relationships” of patients who responded that they had problems with “family relationships” in this study. Therefore, in this study, “family relationships” results do not account for differences in responses by relationship.

Fourth, because this was a questionnaire survey, it was not possible to ask about the details of the problem in the categories of social participation in the BD-checklist 92 that answered, “There was a problem.” Therefore, we did not consider factors preventing social participation in patients with BD. In the future, we would like to clarify the factors that prevent social participation in patients with BD through qualitative research using interviews.

Fifth, because the mean age of the participants was 52 years, fewer patients had school-related problems. It is assumed that a certain number of patients with BD have problems in school life. Therefore, future studies targeting pediatric patients with BD are required.

Finally, the sample size was small. As the medical institutions that agreed to participate in the survey were concentrated in Tokyo and its surrounding suburbs, approximately 80% of the respondents were patients living in Tokyo or its suburbs, which may have limited the generalizability of the results.

### Conclusion

It was found that the greater the restrictions on social participation, the lower the observed QOL. This study showed that the QOL of patients with BD who experienced problems with housework, relationships, and participation in community activities was significantly lower than that of patients with BD who did not experience such problems. In addition, problems with basic interpersonal relationships, such as with friends and family, and limited participation in community activities and ceremonies were associated with worse QOL.

## Article Information

### Conflicts of Interest

None

### Sources of Funding

This work was supported by the JSPS KAKENHI (grant number; 16K04187).

### Author Contributions

We confirm that all authors were involved in the design and conduct of the study, the acquisition of data, or the analysis and interpretation of data. All authors contributed to and approved the manuscript. HT, HK, and TO designed the study; HT, TF, HK, and HO participated in the preparation of the study and mailed the survey forms; HT, HH, KS, and TF analyzed and interpreted the results; HK, TO, and HK assessed the interpretation of the analytical results; HT drafted the manuscript; all authors reviewed the final manuscript.

### Approval by Institutional Review Board (IRB)

IRB approval code issued: 19-058

The name of the institution that granted approval: The Teikyo University Review Board

## Supplement

Supplementary Table
