# Supplementary material for: Relation of Social Participation Restrictions with Worsening Quality of Life in Japanese Patients with Behçet’s Disease: The 36-item Short Form Survey
Source: JMA J. 2024 Nov 18;8(1):151–64. doi: 10.31662/jmaj.2024-0054 (PMC11799724; doi:10.31662/jmaj.2024-0054)
Supplement: Supplementary Table [file 2433-3298-8-1-0151-s001.pdf]

Supplementary table 1 Ten medical institutions that participated in the questionnaire survey

Medical institution (Number of beds)	Recruited sample number
Yokohama City University Hospital (674)	200
St. Marianna University Hospital (955)	130
Nippon Medical School Hospital (877)	60
Teikyo University Hospital (1,074)	45
Hokkaido University Hospital (922)	40
Tokyo Medical University Hospital (904)	40
Kitasato University Hospital (1,185)	20
Nobuhara Hospital (60)	5
Saitama Medical University Hospital (965)	5
Health Sciences University of Hokkaido Hospital (24)	5

Table 2 BD-checklist 92

Body function	
b110	Consciousness functions
b114	Orientation functions
b130	Energy and drive functions
b134	Sleep function
b140	Attention functions
b144	Memory functions
b152	Emotional functions
b156	Perceptual functions
b164	Higher-level cognitive functions
b210	Seeing functions
b230	Hearing functions
b235	Vestibular functions
b250	Taste function
b280	Sensation of pain
b410	Heart functions
b415	Blood vessel functions
b420	Blood pressure functions
b430	Haematological system functions
b435	Immunological system functions
b440	Respiration functions
b4552	Fatiguability
b510	Ingestion functions
b515	Digestive functions
b525	Defecation functions
b620	Urination functions
b640	Sexual functions
b650	Menstruation functions
b710	Mobility of joint functions

b730	Muscle power functions
b735	Muscle tone functions
b765	Involuntary movement functions
b780	Sensations related to muscles and movement functions
b8	Functions of the skin and related structures

Body structures

s110	Structure of brain
s2	The eye, ear and related structures
s410	Structure of cardiovascular system
s5	Structures related to the digestive, metabolic and endocrine systems
s610	Structure of urinary system
s630	Structure of reproductive system
s760	Structure of trunk
s8	Skin and related structures

Activities and Participation

d110	Watching
d115	Listening
d166	Reading
d170	Writing
d210	Undertaking a single task
d240	Handling stress and other psychological demands
d315	Communicating with - receiving - nonverbal messages
d350	Conversation
d430	Lifting and carrying objects
d440	Fine hand use

d450 Walking

d455 Moving around

d465 Moving around using equipment

d470 Using transportation

d475 Driving

d510 Washing oneself

d530 Toileting

d540 Dressing

d550 Eating

d560 Drinking

d570 Looking after one's health

d620 Acquisition of goods and services

d630 Preparing meals

d640 Doing housework

d710 Basic interpersonal interactions

d760 Family relationships

d770 Intimate relationships

d820 School education

d845 Acquiring, keeping and terminating a job

d910 Community life

d920 Recreation and leisure

Environmental factors

e110	Products or substances for personal consumption
e115	Products and technology for personal use in daily living
e120	Products and technology for personal indoor and outdoor mobility and transportation
e125	Products and technology for communication
e150	Design, construction and building products and technology of buildings for public use
e225	Climate
e240	Light
e250	Sound
e310	Immediate family
e320	Friend
e325	Acquaintances, peers, colleagues, neighbours and community members
e330	People in positions of authority
e355	Health professionals
e465	Social norms, practices and ideologies
e540	Transportation services, systems and policies
e560	Media services, systems and policies
e570	Social security services, systems and policies
e575	General social support services
e580	Health services, systems and policies
e590	Labour and employment services, systems and policies

Bold categories are 'Participation' categories
